# Community-level interventions for pre-eclampsia (CLIP) in Mozambique: A cluster randomised controlled trial

**DOI:** 10.1016/j.preghy.2020.05.006

**Published:** 2020-07

**Authors:** Esperança Sevene, Sumedha Sharma, Khátia Munguambe, Charfudin Sacoor, Anifa Vala, Salésio Macuacua, Helena Boene, J. Mark Ansermino, Orvalho Augusto, Cassimo Bique, Jeffrey Bone, Dustin T. Dunsmuir, Tang Lee, Jing Li, Eusébio Macete, Joel Singer, Hubert Wong, Hannah L. Nathan, Beth A. Payne, Mohsin Sidat, Andrew H. Shennan, Corssino Tchavana, Domena K. Tu, Marianne Vidler, Zulfiqar A. Bhutta, Laura A. Magee, Peter von Dadelszen, the CLIP Mozambique Working Group

**Affiliations:** a Centro de Investigação em Saúde da Manhiça (CISM), Rua 12, Cambeve, Manhiça, CP 1929 Maputo, Mozambique; b Faculdade de Medicina, Universidade Eduardo Mondlane, Av. Salvador Allende nr. 702, Maputo, Mozambique; c Department of Obstetrics and Gynaecology, University of British Columbia, Suite 930, 1125 Howe Street, Vancouver V6Z 2K8, Canada; d Direcção Provincial de Saúde, Ministério da Saúde, Av. Eduardo Mondlane n^o^ 1008, CP 264 Maputo, Mozambique; e Centre for International Child Health, University of British Columbia, 305 – 4088 Cambie Street, Vancouver V5Z 2X8, Canada; f Departamento de Ginecologia e Obstetrícia, Hospital Central de Maputo, Av. Agostinho Neto n^o^ 167, CP 1164 Maputo, Mozambique; g Instituto Nacional de Saúde, Ministério da Saúde, Distrito de Marracuene, Estrada Nacional N^o^ 1, Maputo, Mozambique; h Centre for Health Evaluation and Outcome Sciences, Providence Health Care Research Institute, University of British Columbia, 588 – 1081 Burrard Street, St. Paul’s Hospital, Vancouver V6Z 1Y6, Canada; j Department of Women and Children’s Health, School of Life Course Sciences, Faculty of Medicine and Life Sciences, King’s College London, 1 Lambeth Place Road, London SE1 7EH, UK; i Centre for Global Child Health, Hospital for Sick Children, 525 University Avenue, Suite 702, Toronto M5G 2L3, Canada

**Keywords:** Cluster randomized controlled trial, Pregnancy hypertension, Mozambique, Community engagement, Mobile health, Community health worker

## Abstract

**Objectives:**

Pregnancy hypertension is the third leading cause of maternal mortality in Mozambique and contributes significantly to fetal and neonatal mortality. The objective of this trial was to assess whether task-sharing care might reduce adverse pregnancy outcomes related to delays in triage, transport, and treatment.

**Study design:**

The Mozambique Community-Level Interventions for Pre-eclampsia (CLIP) cluster randomised controlled trial (NCT01911494) recruited pregnant women in 12 administrative posts (clusters) in Maputo and Gaza Provinces. The CLIP intervention (6 clusters) consisted of community engagement, community health worker-provided mobile health-guided clinical assessment, initial treatment, and referral to facility either urgently (<4hrs) or non-urgently (<24hrs), dependent on algorithm-defined risk. Treatment effect was estimated by multi-level logistic regression modelling, adjusted for prognostically-significant baseline variables. Predefined secondary analyses included safety and evaluation of the intensity of CLIP contacts.

**Main outcome measures:**

20% reduction in composite of maternal, fetal, and newborn mortality and major morbidity.

**Results:**

15,013 women (15,123 pregnancies) were recruited in intervention (N = 7930; 2·0% loss to follow-up (LTFU)) and control (N = 7190; 2·8% LTFU) clusters. The primary outcome did not differ between intervention and control clusters (adjusted odds ratio (aOR) 1·31, 95% confidence interval (CI) [0·70, 2·48]; p = 0·40). Compared with intervention arm women without CLIP contacts, those with ≥8 contacts experienced fewer primary outcomes (aOR 0·79 (95% CI 0·63, 0·99); p = 0·041), primarily due to improved maternal outcomes (aOR 0·72 (95% CI 0·53, 0·97); p = 0·033).

**Interpretation:**

As generally implemented, the CLIP intervention did not improve pregnancy outcomes; community implementation of the WHO eight contact model may be beneficial.

**Funding:**

The University of British Columbia (PRE-EMPT), a grantee of the Bill & Melinda Gates Foundation (OPP1017337).

## Highlights

• Task-sharing activities to detect and manage pregnancy hypertension can be achieved by CHWs.
• Community engagement activities can achieve a community-driven transport plan.
• Intervention effects may have been masked by incomplete implementation or weak in-facility care.
• Contact intensity analyses support the WHO eight contact antenatal care model.
• Condition-focused community-based interventions without facility strengthening are inadequate.

## 1 Introduction

The maternal mortality ratio (MMR) in Mozambique remains high at 327/100,000 livebirths (2015), despite an average annual decrease of 4·2% from 1990 to 2015 [1]. Most maternal deaths are due to avoidable or preventable causes, and pregnancy hypertension is among the leading causes of death after postpartum haemorrhage and sepsis [2], [3], [4]. Pregnancy hypertension, that complicates 10.9% of pregnancies in the study region [5], is associated with adverse maternal and perinatal outcomes amenable to early identification and timely management [6].

Although efforts have been made to train health personnel in facilities to administer treatment for eclampsia (e.g., magnesium sulphate for eclampsia prevention or treatment [including in primary health centres]) [7], our formative work identified that maternal health in rural Mozambique is influenced by a range of sociocultural factors (e.g., complex gender relations resulting in low autonomy for women to seek care), economic factors (e.g., barriers related to cost of seeking care, and lack of transport other than between-facility ambulance services), and environmental factors (e.g., seasonal variation in access to health facilities) [8], [9], [10].

To address the ‘three delays’ in triage, transport, and treatment [11], and reduce the incidence of associated maternal and perinatal complications, it has been suggested that the identification of cases, intervention and prompt referrals should start at the community level [12]. The Government of Mozambique introduced a community health worker (CHW) programme in 1978 to reach remote populations with health promotion and disease prevention activities. Recently, the programme was revitalised to cover maternity care, including promotion of antenatal and postnatal care visits, promotion of facility-based delivery, identification of warning signs (e.g., oedema), and referral to nearby health facility. However, there is no curriculum content directed at diagnosis or management of pregnancy hypertension [13].

The aim of the Community-Level Interventions for Pre-eclampsia (CLIP) cluster randomised controlled trial (cRCT) in Mozambique was to reduce all-cause, maternal and perinatal mortality and major morbidity by 20% in intervention clusters in Maputo and Gaza Provinces.

## 2 Methods

The full protocol has been published (https://clinicaltrials.gov/ct2/show/NCT01911494; Appendix 1) and ethically approved (Centro de Investigação em Saúde da Manhiça (CISM, CIBS-CISM/038/14), Mozambique National Bioethic Committee (219/CNBS/14) and University of British Columbia (UBC, H12-03497)).

### 2.1 Study setting and trial design

**Fig. 1:**
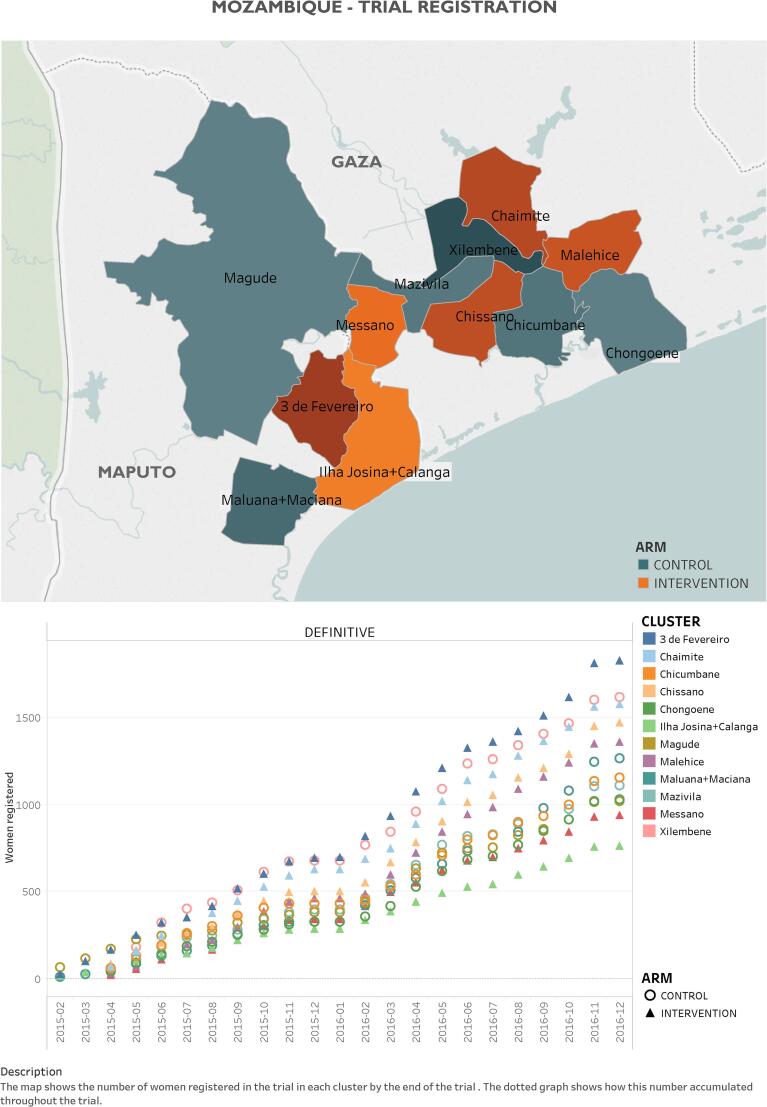
Map of study area and enrollment by cluster.

The CLIP Mozambique trial was one of three independently powered cRCTs (others in Pakistan and India; all NCT01911494), and was conducted in Maputo and Gaza Provinces, southern Mozambique (Fig. 1) with average population per cluster of 24,526 inhabitants (range: 7,499–36,663) [14]. The socioeconomic status varies widely between and within clusters. Generally, households in Gaza possess more assets than those in Maputo province. In Ilha Josina and Calanga, nearly-half of the households are in the poorest wealth quintile [14]. Agriculture is the main occupation for women in this area [14].

A formative 12-month feasibility study delineated baseline characteristics of the study area and barriers and facilitators related to implementation of the intervention [8], [9], [10], [14], [15]. The CLIP Mozambique Trial did not include a pilot phase.

### 2.2 Randomisation

The unit of randomisation (cluster) was the administrative post and all villages and primary health centres (PHCs) within that post (minimum population of 25,000) were included in that cluster; in areas where population size was much lower than 25,000, two administrative posts were joined together to form one cluster. Twelve potential clusters were chosen by the local team, according to similar health care infrastructure, accessibility to the surveillance team, and the absence of conflicting concurrent research activity; four were from Maputo (3 de Fevereiro, Maluana/Maciana, Ilha Josina/Calanga, Magude) and eight from Gaza (Xilembene, Chicumbane, Nhancutse, Chibuto, Macia, Chissano, Mazivila, Messano) Provinces. Restricted, stratified randomisation was undertaken according to population size. There were 21 allocations that met these minimisation criteria; of these, one was selected using a random number sequence generator to assign the six intervention and six control clusters.

### 2.3 Participants

Women of reproductive age were identified based on six-monthly household surveillance that followed written informed consent by the head-of-household. Pregnant women were eligible if they were aged 12–49 years. All participants provided written informed consent; if under 18 years, the participant provided written assent and her guardian, written consent. In intervention and control clusters, consent covered six-monthly household data collection. In intervention clusters, consent also included CHW-provided home visits (see ‘Interventions’). The surveillance team informed the cluster CHWs about eligible and consented pregnant women with registration data. All recruited participants were followed up to satisfy ethical requirements that women complete their postpartum visit.

### 2.4 Interventions

In the intervention arm (six clusters), the CLIP intervention package consisted of both community engagement and CHW-led visits with eligible women in their homes or PHC, with a focus on the detection and management of pregnancy hypertension (Figure S1 and Table S2).

Community engagement was delivered from the time of cluster randomisation until the end of follow-up. Sessions were held with community leaders, women in the communities and their mothers, mothers–in-law, and husbands. Pregnant women and women of reproductive age were engaged during CHW home visits, nurse-led talks at PHCs and community meetings with community activists hired by the trial. Family members also participated in these meetings, usually husbands or partners, mothers-in-law or elders. Cluster-level mobilisers and the CLIP community liaison officer led events for community stakeholder groups (e.g., community leaders, small business entrepreneurs, and neighbourhood secretaries). Fifteen nurses, 48 CHWs, and three community activists were trained to deliver key messages addressing the ‘three delays’ in triage, transport, and treatment, including: (i) awareness of symptoms, signs, and potential consequences of pre-eclampsia and eclampsia; and (ii) birth preparedness and complication-readiness, including prior permissions for care-seeking, savings for obstetric emergencies, and transport planning, if needed. Activities were supported by culturally-appropriate pictograms developed with local input and describing maternal and perinatal risks associated with pregnancy hypertension.

To address the specific transport problem, 77 meetings were held with 665 participants. A community transport scheme was designed to ensure adequate emergency obstetric care-preparedness among pregnant women and communities. A start-up fund was provided by CLIP, based on the anticipated local number of obstetric emergencies. The communities agreed to contribute funds to sustain the scheme post-trial. A committee assigned by the community and composed of three to four influential community members managed the funds.

The CHW-provided contacts were recommended to occur every four-weeks (<28 weeks), fortnightly (28–35 weeks), weekly (36 weeks until birth), within 24 h of birth and on postpartum days 3, 7, and 14. CHW tasks were guided by the tablet-based PIERS On the Move (POM) mobile health (mHealth) application (app) [16], [17] that included miniPIERS (Pre-eclampsia Integrated Estimate of RiSk) time-of-disease risk stratification (Table S2) [18], [19] with pictograms as visual prompts. CHW-provided contacts were from enrolment until six weeks postpartum.

There were 50 CHWs trained to provide the CLIP intervention. These included part of 48 new CHWs hired to address the low coverage of CHWs in the study areas (intervention and control); these hires were in collaboration with UNICEF and the Gaza and Maputo Provincial Directorates that agreed to sustain this workforce post-trial. The CHWs underwent 15 days’ didactic and participatory training in five modules, including: understanding pre-eclampsia and eclampsia and the CLIP protocol, conducting blood pressure (BP) measurement and using the CLIP POM tool, and undertaking skilled basic communication and obtaining informed consent. After study initiation, each CHW received on-the-job training and follow-up until all study procedures were understood and well-implemented, with monthly refresher training.

In brief, the POM directed CHWs to first observe women to rule out emergency conditions (illustrated by pictograms) that would warrant immediate referral to facility. In the absence of emergency conditions, CHWs were directed to measure BP twice, using standardised methods and a semi-automated digital device validated for use in pregnancy (Microlife BP 3AS1-2) [20]; a third measurement was required if either the systolic or diastolic BP differed by more than 10 mmHg between the first two readings. Hypertension and severe hypertension were defined by systolic BP ≥140 mmHg and ≥160 mmHg, respectively; isolated diastolic hypertension did not elicit a response.

The CLIP POM tool stratified women into one of three care pathways: usual ante-/postnatal care, non-urgent referral to a comprehensive emergency obstetric care (CEmOC) or higher facility within 24 h, or urgent referral to a CEmOC or higher facility within 4 h. In addition, women could be recommended to receive community-initiated oral antihypertensive therapy (methyldopa 750 mg for severe hypertension) or 10 g intramuscular magnesium sulphate (for evidence of complicated pre-eclampsia) (Figure S1 and Table S2).

Women in control clusters received routine ANC provided at PHCs by nurses and doctors. The workforce was supplemented by an additional 87 CHWs in control clusters who received basic CHW, but neither BP measurement nor CLIP-specific, training.

At both intervention and control cluster hospitals, facility enhancement (6 sessions) occurred to promote evidence-based care of hypertensive pregnant women.

### 2.5 Adverse event monitoring

Adverse events were monitored in intervention and control clusters with special interest to relative maternal hypotension on arrival at facility (defined as sBP < 110 mmHg) after methyldopa administration in the community; injection site haematoma or infection, either respiratory depression, coma or death after magnesium sulphate administration in the community; transport-related injury (life or limb) or death during transport; ≥20% of women referred to facility being sent back to their communities without follow-up (monitoring community engagement and the CLIP POM). The adverse events are presented by treatment group: number of adverse events (overall and by type), number of women with one or more adverse event(s) (overall and by type).

### 2.6 Procedures for surveillance

In both intervention and control clusters, the CISM Demography Department undertook six-monthly cross-sectional demographic surveys to identify eligible pregnant women and collect baseline and outcome data. In total, 12 field supervisors and 60 field workers received two-weeks’ training. The demography team undertook weekly field monitoring and supervision throughout the trial, and a web-based application (*manhica-dbsync*) was modified for quality control and data management between surveillance forms.

Data collection tools were created iteratively with local investigators, derived from existing validated questionnaires where possible (e.g., WHO 2012 Verbal and Social Autopsy) [5], and translated to Portuguese. Initially women’s demographic characteristics, and past medical and obstetric history were collected, with subsequent six-monthly data collection on care-seeking during pregnancy and pregnancy outcomes. The mobile application synchronised with the CISM servers biweekly, facilitating transfer of information about deceased mothers and babies into the verbal autopsy instruments used by field officers for all maternal and perinatal deaths.

Data were collected on electronic tablets (customised OpenHDS application) to give surveillance staff access to all contemporaneous trial data. Data management protocols ensured data security by encryption, data tracking through user identification numbers and audit trails, and effective data synchronisation between within-cluster devices, the CISM server, and the UBC REDCap (Vanderbilt University, USA) server.

### 2.7 Outcomes

The primary outcome was a composite of all-cause maternal, fetal, and newborn mortality and major morbidity (defined in Panel). Mortality was assessed until 42 and 28 days after birth for mothers and offspring, respectively, and described per 1000 identified pregnancies. Maternal morbidity was not limited to those related to hypertensive pregnancy, as it was possible that the CLIP intervention might favourably influence outcomes in general. Neonatal morbidity reflected problems related to early delivery or delivery of a baby in poor health. All deaths, as well as survived morbidities, were reviewed by an independent panel (two obstetricians, two paediatricians, and one epidemiologist), masked to the cluster of origin, and excluding individuals who cared for the woman or baby under review.

The major secondary outcomes were birth preparedness and complication readiness, delivery in facility able to provide emergency obstetric care, and proportion of facility births. Other outcomes included the impact of the intensity of contacts on the incidence of the primary outcome and its components, gestational age at birth, and mode of delivery.

### 2.8 Sample size

The requirement for 12 clusters over two years was estimated assuming an (i) annual birth rate of 40/1000/year in each cluster; (ii) a baseline incidence of our primary outcome (of one or more of maternal, fetal, or neonatal mortality or major morbidity) of 11·1% in intervention clusters; (iii) an intra-cluster co-efficient (ICC) of 0·002; (iv) a 20% reduction in all cause maternal, fetal, and newborn morbidity and mortality in intervention vs. control clusters; (v) an alpha of 0·05; (vi) 80% power and (vii) 10% loss to follow-up. The sample size was calculated using ‘Iceberg Sim’ software (version 2.0), based on simulations of 5000 Monte Carlo samples based on the input parameters. Power was calculated as the total number of the times within the 5000 samples there was a 20% difference in the primary composite outcome between intervention and control clusters, at p < 0.05. The data upon which the estimates were made were derived from the published rates or were provided by site investigators.

### 2.9 Statistical analysis (full plan, Appendix 2)

All pregnancies, except those of women who withdrew, were included in our primary, intention-to-treat analyses. The unit of analysis was pregnancy, classified as ‘followed-up’ (complete postpartum trial surveillance), ‘lost-to-follow-up’ (estimated date of delivery [EDD] at ≥3 weeks before trial end but without follow-up data), and ‘still-on follow-up’ (EDD < 3 weeks before trial end).

To mitigate potential bias due to differential loss-to and incomplete follow-up, the primary outcome of women who were lost-to, or still-on, follow-up was imputed ten times via multiple imputation by chain equations and Rubin’s rules [21]. Imputation models were based on all primary analysis adjustment factors (see below) and interactions between trial arm and enrolment date (accounting for possible lag in intervention effects). In each imputed data set, the adjusted odds ratio (OR) for the intervention effect was estimated using a multi-level logistic regression including a random intercept term for each cluster. To improve precision, models were adjusted for variables at individual (i.e., age, parity, maternal primary education, previous delivery locations, and husband’s primary education) and cluster-level (i.e., baseline neonatal mortality rate, CHW density, and population density). Sensitivity analysis including only complete cases was conducted with the same adjustment variables.

Further, analogous multi-level logistic regression models were fit to assess the sensitivity of the primary result to various other factors, including adjustment, missing data for a component of the primary outcome (when others were documented), gestational age at birth, and postpartum follow-up to <42 days, as well as cluster-level aggregate analysis. Where sensitivity analyses included imputation, results were pooled (Rubin’s rules) [21]. In an additional planned secondary analysis, we explored within the intervention arm, whether there was a relationship between our primary outcome and the intensity of CLIP contacts, categorised as 0, 1–3, 4–7, or ≥8, to reflect prior and current WHO recommendations for the frequency of antenatal contacts [22]; to account for factors related to the number of POM-guided visit and confounders, the analysis was adjusted for maternal age, basic education, parity, enrolment timing in the trial, and distance from the household to facility.

All analyses were repeated for each component of the primary outcome, albeit without imputation. Secondary and other outcomes were compared by baseline factor-adjusted multi-level models, as above.

Statistical significance (two-sided) was set at p < 0·05 for the primary and p < 0·01 for other analyses, without adjustment for the interim analysis. R statistical software was used throughout.

An interim analysis was undertaken once complete data were received for women making up half of the planned sample size and reviewed by the data safety and monitoring board (DSMB). The stopping rule for both benefit and harm required an observed difference between groups associated with an alpha < 0·001 (power 80%). The DSMB reviewed all reported adverse events for participant safety.

## 3 Results

**Fig. 2:**
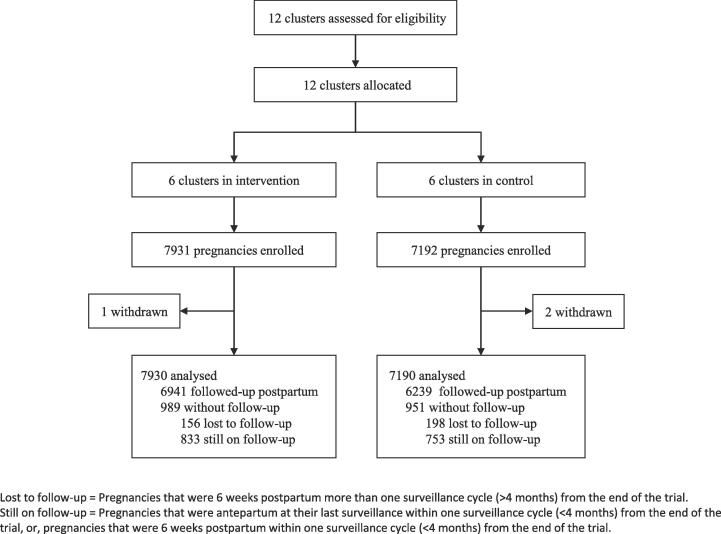
Trial profile – Intervention vs. control allocation clusters.

Between 24 February 2015 to 24 February 2017, 15,013 women (15,123 pregnancies) were recruited in six intervention (7931 pregnancies) and six control (7192 pregnancies) clusters. There were three withdrawals, leaving 7930 pregnancies in intervention and 7190 in control in the primary analysis (Fig. 2).

Clusters in the intervention (vs. control) arm were more remote, as reflected in lower population density (i.e., population/hectare) and healthcare worker density (i.e., number of healthcare workers/10,000 people) (Table 1). Pregnancies in intervention and control clusters were broadly similar at baseline, with most information coming directly from women themselves (Table 1). Women were generally in their early 20 s, married, self-identified as Zionist or Christian, and had received a basic education (as had their partners). Women were generally parous and when so, reported high rates of prior (usually non-CEmOC) facility delivery, stillbirth (12·6%), and neonatal death (6·6%). Associated HIV (21·1%) and malaria (37·5%) were common. Few women (2%) reported multiple pregnancies and most women were enrolled around 25 weeks’ gestation. The maternal mortality ratio (MMR) in the study was 149 per 100,000 livebirths; stillbirths and neonatal deaths occurred at rates of 24·3 and 26·4 per 1000 livebirths, respectively.

**Table 1:** Baseline characteristics.

	Intervention (n = 7930 pregnancies)	Control (n = 7190 pregnancies)
*Clusters*	6	6
Population density (n/ha)*	39·7	52·3
Estimated annual birth rate/cluster	1000	1000
Healthcare worker density (/10,000 population)*†*	2·9	4·4
Neonatal mortality ratio/1000 live births (in last 12 months at baseline)†	10·5 (8·4–11·9)	13·0 (11·0–18·2)
Households	7142	6664
*Enrolled pregnancies*	7930	7190
Nature of respondent was woman herself	6774 (85·4%)	6198 (86·2%)
Maternal age (year)*	23 (19–30)	23 (19–30)
Married*†*	5443 (68·6%)	4470 (62·2%)
*Religion*		
Zion/Siao/Christian Protestant/Anglican/Undetermined Christian	4294 (54·1%)	3705 (51·5%)
Catholic	932 (11·8%)	715 (9·9%)
Other	2349 (29·6%)	2690 (37·4%)
Women with ≥5 years of schooling*	4563 (57·5%)	4946 (68·8%)
Husbands with ≥5 years of schooling*†*	2795 (51·4%)	2626 (58·7%)
HIV-positive	1701 (21·5%)	1489 (20·7%)
Malaria†	3255 (41·1%)	2414 (33·6%)
*Obstetric history*		
Parous*	5495 (69·3%)	4876 (67·8%)
Parity	2·0 (0·0–3·0)	2·0 (0·0–3·0)
*Amongst previously pregnant women*		
Had previous stillbirth(s)	769 (13·7%)	561 (11·3%)
Had previous neonatal death(s)	393 (7·3%)	302 (6·3%)
*Delivery location in previous pregnancy*		
Home*†*	902 (16·0%)	593 (12·0%)
CEmOC (hospitals)	754 (13·4%)	782 (15·8%)
Non-CEmOC facility	3666 (65·2%)	3326 (67·1%)
ANC care sought*†*	3873 (68·9%)	2827 (57·0%)
*Current pregnancy*		
Gestational age at enrolment (week)	24·8 (18·9–31·6)	26·4 (20·4–33·0)
Multiple pregnancy	186 (2·3%)	134 (1·9%)

Data presented as median (interquartile range) or number (%). *Variables used as adjustment factors in analyses, chosen *a priori*. †Variable used as adjustment factor in analyses, chosen following review of baseline data prior to knowledge of outcomes.

ANC = antenatal care. HIV = human immunodeficiency virus. CEmOC = comprehensive emergency obstetric care.

There were 4243 community engagement sessions for a median (IQR) of 811 (464, 910) sessions/cluster (Table S3). There were 19,219 participants, including women of reproductive age (2338), pregnant women (7762), mothers and mothers-in-law (1331), husbands and partners (1461), and community stakeholders (1 0 6) (Table S3).

CHW coverage (per 10,000 population served) varied between clusters (range: 1·7% (3 De Fevereiro, Maputo) to 6·8% (Malehice, Gaza)). The 48 CHWs in the intervention arm conducted 28,514 home-based CLIP contacts; 4781 women (60.3%) received at least one contact, for a median [IQR] of six (3, 8) contacts per pregnancy when received. The contact intensity compliance target was met in 2678 (56·0%) pregnancies. Most contacts were antenatal (71·5%). Compliance with BP measurement at all contacts (99·2%) and proteinuria measurement at the first and all subsequent contacts during which hypertension was detected (97·7%) was virtually uniform (Table S3).

POM-guided referrals, whether non-urgent (3·1% of pregnancies) or urgent (2·4% of pregnancies), were accepted 72·3% and 61·8% of the time, respectively. The most common reason for urgent referral was severe hypertension (30/114 visits, 26·3%), followed by absence of fetal movements in preceding 12 h (25/114 visits, 21·9%). Intramuscular magnesium sulphate was recommended at 35 (0·1%) visits and accepted 51·4% of the time. Oral methyldopa was recommended in 35 (0·1%) visits and accepted 71·4% of the time (Table S3). Details of the CLIP indications for referral can be found in Table S2.

Although less than half of women received ≥4 routine antenatal clinic visits, more women did so in intervention (48·6%) vs. control (42·5%) clusters (aOR 1·57 [99% CI 0·97, 2·52]; p = 0·016).

The composite primary outcome occurred in ≈16% of pregnancies, driven primarily by major maternal morbidity (≈10%), rather than either fetal or newborn mortality (≈5%) or neonatal morbidity (≈4%) (Table 2). Major maternal morbidity was nearly 68-fold more common than maternal mortality (1·5/1000 identified pregnancies), due most commonly to antepartum haemorrhage (4·6%), stroke (3·3%), obstetric sepsis (3·2%), or possible eclampsia (reported seizures with hypertension) (0·2%). Major neonatal morbidity was due primarily to seizure (1·3%), coma (0·9%), feeding difficulty (0·9%), lethargy (0·9%), and hypothermia (0·9%), with the others occurring at ≤0·5%.

**Table 2:** Primary outcomes.

	Intervention (n = 7930 pregnancies)	Control (n = 7190 pregnancies)	Adjusted OR (95% CI)*	p-value
Pregnancies with postpartum surveillance	6941 (87·5%)	6239 (86·8%)		
Infants	7099	6355		
*Composite primary outcome†*	1246 (15·7%)	1172 (16·3%)	1·31 (0·70-2·48)	0·40
Maternal mortality	15 (0·2%)	7 (0·1%)	1·98 (0·60-6·56)	0·26
Maternal morbidity	731 (9·2%)	688 (9·6%)	1·35 (0·59-3·10)	0·48
(including maternal deaths)				
Antepartum haemorrhage	417 (5·3%)	284 (3·9%)		
Stroke	240 (3·0%)	263 (3·7%)		
Obstetric sepsis	206 (2·6%)	280 (3·9%)		
Maternal coma	139 (1·8%)	98 (1·4%)		
Interventions for major postpartum haemorrhage	69 (0·9%)	148 (2·1%)		
Seizure	72 (0·9%)	99 (1·4%)		
Fistula	61 (0·8%)	92 (1·3%)		
Disseminated intravascular coagulation	53 (0·7%)	20 (0·3%)		
Cardiopulmonary resuscitation	42 (0·5%)	28 (0·4%)		
Blood transfusion	38 (0·5%)	31 (0·4%)		
Mechanical ventilation	36 (0·5%)	31 (0·4%)		
Dialysis	0 (0·0%)	5 (0·1%)		
Perinatal mortality and late neonatal death	411 (5·2%)	331 (4·6%)	1·23 (0·99-1·54)	0·06
Stillbirth	196 (2·5%)	162 (2·3%)	1·34 (1·01-1·79)	0·04
Early neonatal death	182 (2·3%)	149 (2·1%)	1·08 (0·82-1·43)	0·56
Late neonatal death	36 (0·5%)	23 (0·3%)	1·47 (0·72-3·00)	0·30
Neonatal morbidity	243 (3·1%)	321 (4·5%)	0·99 (0·32-3·07)	0·98
Seizure	88 (1·1%)	115 (1·6%)		
Coma	50 (0·6%)	93 (1·3%)		
Feeding difficulty	61 (0·8%)	74 (1·0%)		
Lethargy	48 (0·6%)	83 (1·2%)		
Other central nervous system-related morbidity	10 (0·1%)	7 (0·1%)		
Hypothermia	30 (0·4%)	100 (1·4%)		
Umbilical cord infection	57 (0·7%)	23 (0·3%)		
Bleeding	40 (0·6%)	28 (0·4%)		
Jaundice	32 (0·4%)	27 (0·4%)		
Breathing difficulty	22 (0·3%)	33 (0·5%)		
Skin infection	0 (0·0%)	4 (0·1%)		

Data presented as number (%) or number only. *Adjusted odds ratio presented as odds ratio (95% confidence interval); adjusted for individual-level factors (maternal age, parity, maternal education, marital status, husband’s education, delivery location), and cluster-level factors (population density, baseline study neonatal mortality rate, healthcare worker density). † Defined as one/more of maternal morbidity or mortality, stillbirth, neonatal mortality, or neonatal morbidity.

OR = odds ratio.

The composite primary outcome did not differ in intervention (vs. control) arms (aOR 1·31 [0·70–2·48]; p = 0·40) in the primary (Table 2) or planned sensitivity analyses (Table S4). The clusters had quite variable primary outcome rates (ICC of 0·073). The other outcomes did not differ between groups (Table 3). A small proportion of this generally late-booking cohort’s pregnancies ended in miscarriage (1·5%). Most women gave birth in non-CEmOC facilities, by spontaneous vaginal delivery. About 20% of births were preterm.

**Table 3:** Secondary, safety, and other outcomes.

	Intervention (n = 7930 pregnancies)	Control (n = 7190 pregnancies)	Adjusted OR (99% CI)*(	p-value
*Secondary outcomes*				
Birth preparedness and complication readiness*†*	3462 (43·7%)	3569 (49·6%)	0·91 (0·47, 1·77)	0·72
Proportion of facility births	5803 (67·3%)	5339 (74·2%)	0·85 (0·28, 2·61)	0·71
Birth at a CEmOC facility	897 (11·3%)	936 (13·0%)	0·85 (0·27, 2·62)	0·70
*Safety outcomes*				
SAEs unrelated to intervention	0 (0·0%)	0 (0·0%)	NA	NA
Adverse events				
Transport-related injury or death	0/158 (0·0%)	NA	NA	NA
Injection site haematoma/infection after community administration of IM MgSO4	0/13	NA	NA	NA
Injection site complications after any administration of IM MgSO_4_	NA	NA	NA	NA
Respiratory depression, coma or death during transport following in-community MgSO_4_	NA	NA	NA	NA
Maternal sBP < 110 mmHg on facility arrival following in-community methyldopa	NA	NA	NA	NA
*Other outcomes*				
Deliveries (all birth outcomes)	6928	6228	NA	NA
Deliveries (excluding miscarriage)	6825	6132	NA	NA
Miscarriage	107 (1·5%)	98 (1·6%)	0·90 (0·45, 1·80)	0·69
Live births	6636 (95·8%)	5976 (96·0%)	0·88 (0·61, 1·21)	0·34
Gestational age at delivery (week)	39·3 (37·1–41·0)	39·3 (37·1–41·0)		0·90
Deliveries < 37 weeks	1426 (20·6%)	1295 (20·8%)	0·94 (0·70, 1·25)	0·56
Deliveries < 34 weeks	588 (8·5%)	557 (8·9%)	0·90 (0·73, 1·12)	0·21
Missing	832 (12·0%)	775 (12·4%)	NA	NA
Mode of delivery (exclude miscarriage and terminations)			0·78 (0·57, 1·05)	0·03
Spontaneous vaginal	5209 (76·3%)	4577 (74·6%)	–	–
Assisted vaginal	1369 (20·1%)	1289 (21·0%)	–	–
Caesarean delivery	239 (3·5%)	260 (4·2%)	–	–

Data presented as median (interquartile range) or number (%) or number only. *Odds ratio adjusted for individual-level (i.e., maternal age, parity, maternal primary education, previous delivery locations, and husband’s primary education) and cluster-level (i.e., baseline neonatal mortality rate and population density) characteristics). †Birth preparedness and complication readiness was defined as an answer to ALL three of the following: 1) arranged for transport, 2) obtained prior permission to seek emergency care, and 3) saved money for obstetric care.

OR = odds ratio. CEmOC = comprehensive emergency obstetric care. sBP = systolic blood pressure.

There were no serious adverse events, particularly neither intervention arm transport-related injuries or death nor haematomas or infections after community administration of intramuscular magnesium sulphate (Table 3).

Within intervention clusters, women who received at least eight POM guided contacts experienced fewer adverse outcomes compared with women who received no contacts (11.0% vs 19.9%, aOR 0.79 [95% CI 0·63, 0·99]; p = 0·041). This decrease was consistent for all components of the outcome, but most obvious for maternal morbidity (5.7% vs 13.1%, aOR 0.74 [95% CI 0·55, 1·01]; p = 0·056) (Table 4). Women with 1–3 or 4–7 POM-guided contacts did not have apparent benefit compared with those without POM contacts (Table 4). Rates of neonatal death and stillbirth were higher in both the 1–3 visit group (9.9% vs 5.1% aOR 2.15 [95% CI 1·58, 2·91]; p=<0.001) and the 4–7 visit group (7.2% vs 5.1% aOR 1.48 [95% CI 1·13, 1·95]; p = 0·005) compared with those receiving no visits.

**Table 4:** Relationship between intensity of POM-guided CLIP contacts and the primary outcome.

Outcomes	Number of POM-guided visits										
	0 visits		1–3 visits			4–7 visits			≥8 visits		
	Event rate	Adjusted OR (95% CI)†	Event rate	Adjusted OR (95% CI)†	P	Event rate	Adjusted OR (95% CI)†	p	Event rate	Adjusted OR (95% CI)†	p
Primary outcome‡	543 (19·9%)	*Reference*	204 (22·4%)	1·42 (1·15, 1·76)	0·001	334 (18·6%)	1·18 (0·99, 1·40)	0·07	152 (11·0%)	0·79 (0·63, 0·99)	0·041
Maternal outcome	361 (13·3%)	*Reference*	110 (12·2%)	1.06 (0·81, 1·39)	0·67	185 (10·4%)	1·02 (0·81, 1·28)	0·88	78 (5·7%)	0·72 (0·53, 0·97)	0·033
*Maternal mortality*	7 (0·3%)	*Reference*	2 (0·2%)	inestimable	–	4 (0·2%)	Inestimable	–	0 (0·0%)	inestimable	–
*Maternal morbidity*	356 (13·1%)	*Reference*	108 (12·0%)	1.09 (0·83, 1·43)	0·54	183 (10·2%)	1·04 (0·83, 1·31)	0·74	78 (5·7%)	0·74 (0·55, 1·001)	0·056
Fetal or neonatal adverse outcome	259 (9·5%)	*Reference*	116 (12·9%)	1·56 (1·21, 2·03)	0·001	191 (10·7%)	1·27 (1·02, 1·59)	0·036	87 (6·3%)	0·81 (0·61, 1·09)	0·16
*Stillbirth*	60 (2·2%)	*Reference*	44 (4·8%)	2·40 (1·54, 3·74)	<0·001	64 (3·6%)	1·76 (1·19, 2.62)	0·005	28 (2·1%)	0·94 (0·57, 1·12)	0·16
*Neonatal mortality*	79 (3·0%)	*Reference*	46 (5·3%)	1·87 (1·24, 2·80)	0·003	64 (3·7%)	1·25 (0·87, 1·81)	0·23	28 (2·1%)	0·69 (0·43, 1·10)	0·12
*Neonatal morbidity*	135 (5·0%)	*Reference*	37 (4·1%)	0·99 (0·67, 1·50)	0·96	677 (3·8%)	0·92 (0·66, 1·30)	0·65	36 (2·6%)	0·91 (0·60, 1·39)	0·67

CI = confidence interval. OR = odds ratio.

## 4 Discussion

In this cRCT of community-level interventions for pre-eclampsia, our package of uniformly evidence-based community engagement and mHealth app-guided CHW-provided community-level visits did not decrease a composite outcome of maternal, fetal, or newborn mortality or major morbidity (aOR 1·31 [95% CI 0·70, 2·48]), notwithstanding the outcome rate variation between clusters being more different (ICC 0·073) than anticipated (ICC 0·002), reducing our statistical power.

The finding of a positive effect of contact intensity of at least eight contacts provides data to support the current WHO recommendations [22]. While the WHO recommendations are based on improved perinatal outcomes, our findings (vs. 0 contacts) imply that maternal benefit prevailed in this study (Table 4). Most women with 0 contacts lived closer to facilities than those with 1–3 contacts; women with 0 contacts and women with 1–3 contacts both had 50% higher rates of fetal and neonatal mortality. We were unable to determine how living in a peri-setting urban setting was associated with improved outcomes, but have assumed that our adjustment for distance may have been insufficient to correct for any effect, and that peri-urban women tend to be more affluent than women living remotely and have generally better health status due to access to greater food security, dietary diversity and lower risk from malaria. Compared with women who received 1–3 contacts, other than maternal death and neonatal morbidity, all components of the primary outcome were significantly reduced with at least eight contacts; this effect was noted in both of the other CLIP trials in India and Pakistan (in Pakistan from at least four contacts).

The study design and delivery of the intervention had several strengths, particularly given the context of the trial.

To our knowledge, this is the first and largest community-level study of pregnancy hypertension in Mozambique. We engaged a wide array of stakeholders, including community leaders, non-governmental organisations, funders, civil society organisations, healthcare workers, and sub-national and national directorates in the trial and preceding feasibility study. There is a legacy of two aspects of community infrastructure: (i) the community-driven and developed novel transport scheme; and (ii) the additional 48 CHWs who were recruited and trained to strengthen primary health care, in co-ordination with the national programme and the Maputo and Gaza Provincial Directorates.

CHWs (men and women) were accepted by their communities and effectively delivered a complex intervention without adverse events, despite their low education level and lack of previous training either to measure BP, proteinuria, or pulse oximetry [15], or to administer medications in pregnancy. This has important implications for additional task-sharing to strengthen access to primary and maternity health care, as they already administer of oral misoprostol to prevent postpartum haemorrhage and injectable contraception in the community [23]. CHWs must cover large areas (of 5–8 km) around health facilities, are sustained by external partners and restrictions on spending limit governmental ability to regularise their employment.

Also, we report unique population-level estimates of maternal, fetal and neonatal outcomes, as well as data on care-seeking practices and baseline characteristics, in Mozambique. The study involved over 15,000 women, very few of whom were lost-to-follow-up, even in remote villages with little or only seasonal access to roads and where vital registration and health information systems are weak and inadequate. These data facilitated consideration of individual-level characteristics and greater precision in our analytic model. We observed a MMR of 149, which is lower than the 2015 national estimate of 327 [1]; however, Maputo and Gaza provinces have stronger health infrastructure than other areas of the country, particularly the north. Our MMR is similar to that recorded in our baseline study (205) [14]. Our incidence of stillbirth (per 1000 livebirths) of 24·3 is slightly higher than the 2015 national estimate of 20·7, despite use of the same definition [24]. This may reflect underreporting in other datasets due to cultural norms and beliefs prohibiting disclosure of stillbirths [25], [26]. Of note, misclassification of stillbirths as neonatal deaths is unlikely, as our estimate of neonatal mortality (26·4 per 1000 livebirths) is similar to the 2015 national, population-level estimate (25·5), and to 2011 regional rates (population-level demographic health survey (34–37)) [24], [27].

Previously published estimates of major maternal and neonatal morbidity are limited, and not population-based. Our reported estimates of antepartum haemorrhage were disproportionately higher in the intervention arm (5·3% vs. 3·9%, respectively). The difference could be attributed to the sensitisation of women in the intervention arm to report any signs of significant vaginal bleeding to the CHWs, a message reinforced via pictograms and on the POM visits. Given the prevalence of malaria, it is possible that many instances of coma reported in our study (incidence = 3·2%) were related to cerebral malaria (case fatality rate of 50%) [3]. While measures of self-reported maternal, fetal, and neonatal outcomes have been validated in other studies [28], [29], we cannot exclude the possibility of misreporting in our study. We were unable to use physical examination or chart review to confirm diagnoses; we attempted to minimise misreporting by medical review of collected data masked to group allocation, for each primary outcome.

Despite careful consideration of intervention design, the impact of the CLIP intervention could have been limited as we responded solely to systolic (and not the predominant diastolic [5]) hypertension to trigger referral to facility and antihypertensive therapy. The rationale for this was that systolic BP measurement can be undertaken using a sphygmomanometer, cuff, and radial pulse palpation; however, this is now of less concern given the availability of a low-cost validated BP device [20].

We aimed to work within the existing health care system infrastructure to not create a parallel health system, but this meant that the delivery of our intervention was not immune to some of the systemic shortcomings in the Mozambican health system. For instance, in the CLIP trial, the CHWs followed the national guidelines for referral and, as such, were required to refer the women to the nearest health facility, even if that were a PHC unable to manage her complications. In addition, our intervention did not include facility-based care that is challenged in Mozambique by scarce human resources, lack of equipment, and stock outs of essential medication, including magnesium sulphate. When relevant, women from both trial arms were also referred to common secondary facilities, and the quality of care delivered in the facilities may have masked an effect of community-level identification and treatment.

It is possible that the relationship between the CLIP intervention and outcomes was moderated in part by women’s underlying health status, particularly given the high prevalence of HIV infection (≈20%) and malaria (≈40%) in our study population. Of course, there may have been other interactions between participant characteristics and context and the intervention, that mediated the relationship with outcome.

In conclusion, in the CLIP Mozambique Trial, community-level interventions for pre-eclampsia were achieved by CHW. The lack of a significant effect on maternal, fetal, or newborn mortality or major morbidity may be due to the small number of clusters or deficiencies in facility-based care. The study suggests that single condition-focussed community-based interventions, without strengthening quality facility care and referral systems, are inadequate to achieve desired improvements in pregnancy outcomes.

## Declaration of Interests

We declare no competing interests. BAP, LAM and PvD acknowledge that the intellectual property related to the miniPIERS prediction model used in the CLIP trials was transferred in its entirety from the University of British Columbia to them, among other inventors, prior to the trial. They have no financial benefit from the use of the model based on the transfer.

## Appendix A Supplementary data

Supplementary data 3

Supplementary data 4 Figure S1. The CLIP intervention.

## References

[1] Kassebaum N.J., Barber R.M., Bhutta Z.A., Dandona R., Dandona L., Gething P.W. (2016). Global, regional, and national levels of maternal mortality, 1990–2015: a systematic analysis for the Global Burden of Disease Study 2015. Lancet.

[2] Menéndez C., Romagosa C., Ismail M.R., Carrilho C., Saute F., Osman N. (2008). An autopsy study of maternal mortality in Mozambique: the contribution of infectious diseases. PLoS Med..

[3] David E., Machungo F., Zanconato G., Cavaliere E., Fiosse S., Sululu C. (2014). Maternal near miss and maternal deaths in Mozambique: a cross-sectional, region-wide study of 635 consecutive cases assisted in health facilities of Maputo province. BMC Pregnancy Childbirth.

[4] Bailey P.E., Keyes E., Moran A.C., Singh K., Chavane L., Chilundo B. (2015). The triple threat of pregnancy, HIV infection and malaria: reported causes of maternal mortality in two nationwide health facility assessments in Mozambique, 2007 and 2012. BMC Pregnancy Childbirth.

[5] Magee L., Sharma S., Nathan H., Adetoro O., Bellad M., Goudar S. (2019). The incidence of pregnancy hypertension in India, Pakistan, Mozambique, and Nigeria: a prospective population-level analysis. PLoS Med..

[6] von Dadelszen P., Peter L.A. Magee (2016). Preventing deaths due to the hypertensive disorders of pregnancy. Best Practice Res.: Clinical Obstet. Gynaecol..

[7] Duley L., Gülmezoglu A.M., Henderson-Smart D.J., Chou D. (2010). Magnesium sulphate and other anticonvulsants for women with pre-eclampsia. Cochrane Database Syst .Rev..

[8] K. Munguambe, H. Boene, M. Vidler, C. Bique, D. Sawchuck, T. Firoz, et al. Barriers and facilitators to health care seeking behaviours in pregnancy in rural communities of southern Mozambique. Reprod. Health 13 (Suppl 1(S1)) (2016) 31. 10.1186/s12978-016-0141-0 PMC4943506 27356968

[9] Makanga P.T., Schuurman N., Sacoor C., Boene H.E., Vilanculo F., Vidler M. (2017). Seasonal variation in geographical access to maternal health services in regions of southern Mozambique. Int. J. Health Geogr..

[10] Firoz T., Vidler M., Makanga P.T., Boene H., Chiaú R., Sevene E. (2016). Community perspectives on the determinants of maternal health in rural southern Mozambique: a qualitative study. Reprod. Health.

[11] Thaddeus S., Maine D. (1994). Too far to walk: maternal mortality in context. Soc. Sci. Med..

[12] Jamisse L., Songane F., Libombo A., Bique C., Faúndes A. (2004). Reducing maternal mortality in Mozambique: challenges, failures, successes and lessons learned. Int. J. Gynaecol. Obstet..

[13] B.G. Chilundo, J.L. Cliff, A.R. Mariano, D.C. Rodríguez, A. George. Relaunch of the official community health worker programme in Mozambique: is there a sustainable basis for iCCM policy? Health Policy Plan. 30 (suppl 2) (2015) ii54–64. 10.1093/heapol/czv036 PMC4625760 26516151

[14] Sacoor C., Payne B., Augusto O., Vilanculo F., Nhacolo A., Vidler M. (2018). Health and socio-demographic profile of women of reproductive age in rural communities of southern Mozambique. PLoS ONE.

[15] Boene H., Vidler M., Augusto O., Sidat M., Macete E., Menéndez C. (2016). Community health worker knowledge and management of pre-eclampsia in southern Mozambique. Reprod. Health.

[16] Lim J., Cloete G., Dunsmuir D.T., Payne B.A., Scheffer C., von Dadelszen P. (2015). Usability and feasibility of PIERS on the Move: an mHealth app for pre-eclampsia triage. JMIR mHealth uHealth.

[17] Dunsmuir D.T., Payne B.A., Cloete G., Petersen C.L., Gorges M., Lim J. (2014). Development of mHealth applications for pre-eclampsia triage. IEEE J. Biomed. Health Inform..

[18] Payne B.A., Hutcheon J.A., Dunsmuir D., Cloete G., Dumont G., Hall D. (2015). Assessing the incremental value of blood oxygen saturation (SpO2) in the miniPIERS (pre-eclampsia Integrated estimate of RiSk) risk prediction model. J. Obstet. Gynaecol. Can..

[19] Payne B.A., Hutcheon J.A., Ansermino J.M., Hall D.R., Bhutta Z.A., Bhutta S.Z. (2014). A risk prediction model for the assessment and triage of women with hypertensive disorders of pregnancy in low-resourced settings: the miniPIERS (Pre-eclampsia Integrated Estimate of RiSk) multi-country prospective cohort study. PLoS Med..

[20] Nathan H.L., de Greeff A., Hezelgrave N.L., Chappell L.C., Shennan A.H. (2015). An accurate semiautomated oscillometric blood pressure device for use in pregnancy (including pre-eclampsia) in a low-income and middle-income country population: the Microlife 3AS1-2. Blood Press Monit..

[21] Rubin D.B. (1987). Wiley Online Library. Multiple imputation for nonresponse in surveys.

[22] World Health Organization. WHO recommendations on antenatal care for a positive pregnancy (2016). Available at: https://apps.who.int/iris/bitstream/handle/10665/250796/9789241549912-eng.pdf;jsessionid=3B5FE32AE497017E57AE2C7371169A8C?sequence=1 (accessed July 2019). 28079998

[23] Jacinto A., Mobaracaly M.R., Ustáb M.B., Bique C., Blazer C., Weidert K. (2016). Safety and acceptability of community-based distribution of injectable contraceptives: a pilot project in Mozambique. Glob. Health Sci. Pract..

[24] Wang Y., Wang H., Wang L., Bhutta Z.A., Coates M.M., Coggeshall M. (2016). Global, regional, national, and selected subnational levels of stillbirths, neonatal, infant, and under-5 mortality, 1980–2015: a systematic analysis for the Global Burden of Disease Study 2015. Lancet.

[25] Zakar M.Z., Zakar R., Mustafa M., Jalil A., Fischer F. (2018). Underreporting of stillbirths in Pakistan: perspectives of the parents, community and healthcare providers. BMC Pregnancy Childbirth.

[26] Kiguli J., Namusoko S., Kerber K., Peterson S., Waiswa P. (2015). Weeping in silence: community experiences of stillbirths in rural eastern Uganda. Glob. Health Action.

[27] Ministerio da Saude - MISAU/Moçambique, Instituto Nacional de Estatística - INE/Moçambique and ICF International. Moçambique Inquérito Demográfico e de Saúde 2011. Calverton, Maryland, USA: MISA/Moçambique, INE/Moçambique and ICF International. 2011. Available at: https://dhsprogram.com/publications/publication-fr266-dhs-final-reports.cfm. (accessed June 2018).

[28] Souza J.P., Cecatti J.G., Pacagnella R.C., Giavarotti T.M., Parpinelli M.A., Camargo R.S. (2016). Development and validation of a questionnaire to identify severe maternal morbidity in epidemiological surveys. Reprod. Health.

[29] Ronsmans C., Achadi E., Cohen S., Zazri A. (1997). Women's recall of obstetric complications in South Kalimantan, Indonesia. Stud. Fam. Plann..

