# Supplementary material for: Community-level interventions for pre-eclampsia (CLIP) in Mozambique: A cluster randomised controlled trial
Source: Pregnancy Hypertens. 2020 Jul;21:96–105. doi: 10.1016/j.preghy.2020.05.006 (PMC7471842; doi:10.1016/j.preghy.2020.05.006)
Supplement: Supplementary data 3 [file mmc3.docx]

**Supplemental tables**

- Table S1: CLIP Mozambique Working Group
- Table S2: CLIP triggers and treatment
- Table S3: Intervention
- Table S4: Sensitivity analyses for primary outcome

**Table S1 CLIP Mozambique Working Group**

| **CLIP Mozambique Working Group for appearance on PubMed** | |
| --- | --- |
| **First and middle names** | **Last names** |
| Felizarda | Amose |
| Ana Ilda | Biz |
| Rogério | Chiaú |
| Silvestre | Cutana |
| Paulo | Filimone |
| Marta | Macamo |
| Sónia | Maculuve |
| Ernesto | Mandlate |
| Analisa | Matavele |
| Sibone | Mocumbi |
| Dulce | Mulungo |
| Zefanias | Nhamirre |
| Ariel | Nhancolo |
| Cláudio | Nkumbula |
| Vivalde | Nobela |
| Rosa | Pires |
| Faustino | Vilanculo |
| Rahat N | Qureshi |
| Sana | Sheikh |
| Zahra | Hoodbhoy |
| Imran | Ahmed |
| Amjad | Hussain |
| Javed | Memon |
| Farrukh | Raza |
| Mrutunjaya B | Bellad |
| Shivaprasad S | Goudar |
| Ashalata A | Mallapur |
| Shashidhar G | Bannale |
| Umesh S | Charantimath |
| Keval S | Chougala |
| Richard J | Derman |
| Vaibhav B | Dhamanekar |
| Narayan V | Hoonungar |
| Anjali M | Joshi |
| Namdev A | Kamble |
| Chandrasekhar | Karadiguddi |
| Geetanjali M | Katageri |
| Avinash J | Kavi |
| Gudadayya S | Kengapur |
| Bhalachandra S | Kodkany |
| Uday S | Kudachi |
| Sphoorthi S | Mastiholi |
| Geetanjali I | Mungarwadi |
| Umesh Y | Ramadurg |
| Amit P | Revankar |
| Olalekan O | Adetoro |
| John O | Sotunsa |
| Sharla K | Drebit |
| Chirag | Kariya |
| Mansun | Lui |
| Diane | Sawchuck |
| Ugochi V | Ukah |
| Mai-Lei | Woo Kinshella |
| Shafik | Dharamsi |
| Guy A | Dumont |
| Tabassum | Firoz |
| Ana Pilar | Betrán |
| Susheela M | Engelbrecht |
| Veronique | Filippi |
| William A | Grobman |
| Marian | Knight |
| Ana | Langer |
| Simon A | Lewin |
| Gwyneth | Lewis |
| Craig | Mitton |
| Nadine | Schuurman |
| James G | Thornton |
| France | Donnay |
| Kelly | Pickerill |
| **CLIP Trial Working Group** | |
| Esperança Sevene, Eusébio Macete, Khátia Munguambe, Charfudin Sacoor, Anifa Vala, Helena Boene, Felizarda Amose, Rosa Pires, Zefanias Nhamirre, Marta Macamo, Rogério Chiaú, Analisa Matavele, Faustino Vilanculo, Ariel Nhancolo, Silvestre Cutana, Ernesto Mandlate, Salésio Macuacua, Quinhas Fernandes, Rosa Marlene Cuco, Cassimo Bique, Sibone Mocumbi, Emília Gonçálves, Sónia Maculuve, Ana Ilda Biz, Dulce Mulungo, Orvalho Augusto, Paulo Filimone, Vivalde Nobela, Corsino Tchavana, Cláudio Nkumbula  Jeffrey Bone, Dustin T Dunsmuir, Sharla K Drebit, Chirag Kariya, Mai-Lei Woo Kinshella, Tang Lee, Jing Li, Mansun Lui, Beth A Payne, Kelly Pickerill, Diane Sawchuck, Sumedha Sharma, Domena K. Tu, Marianne Vidler, Ugochi V Ukah, Laura A Magee, Peter von Dadelszen | |
| **CLIP Trial Adjudication Committee** | |
| Nafissa Osman, Cassimo Bique, Natercia Fernandes, Betuel Sigauque Raquel Gonzalez | |
| **CLIP Trial Steering Committee** | |
| J Mark Ansermino, Ana Pilar Betrán, Richard Derman, Shafik Dharamsi, France Donnay, Sharla Drebit, Guy Dumont, Susheela M. Engelbrecht, Veronique Fillipi, Tabassum Firoz, William Grobman, Marian Knight, Ana Langer, Simon Lewin, Gwyneth Lewis, Craig Mitton, Nadine Schuurman, Andrew H Shennan, Joel Singer, Jim Thornton, Hubert Wong | |
| **CLIP Trial Executive Committee** | |
| Olalekan Adetoro, Mrutunjaya M Bellad, Zulfiqar Bhutta, Peter von Dadelszen, Shivaprasad S Goudar, Jerker Liljestrand, Laura A Magee, Ashalata Mallapur, Khátia Munguambe, Beth Payne, Rahat Qureshi, Charfudin Sacoor, Esperança Sevene, Sumedha Sharma, John Obafemi Sotunsa, Marianne Vidler | |
| **CLIP Data Safety and Monitoring Board (DSMB**) | |
| Romano Nkumbwa Byaruhanga, Brian Darlow, Eileen Hutton, Mario Merialdi, Lehana Thabane | |

**Table S2: CLIP triggers and treatment**

|  | **Urgent transport** | **MgSO_4_†**  5g IM in each buttock | **Methyldopa‡** 750mg po | **Non-urgent transport** |
| --- | --- | --- | --- | --- |
| **Maternal risk triggers** |  |  |  |  |
| **sBP ≥160mmHg** (consistent with severe pre-eclampsia | ● | ● | ● |  |
| **Unconsciousness*** | ● |  |  |  |
| With sBP ≥160mmHg | ● | ● | ● |  |
| **Recent stroke or seizure** | ● | ● |  |  |
| With sBP ≥160mmHg | ● | ● | ● |  |
| **Significant vaginal bleeding** | ● |  |  |  |
| With sBP ≥140mmHg (presumed abruption and severe pre-eclampsia) | ● | ● |  |  |
| **miniPIERS predicted probability ≥25%** | ● | ● |  |  |
| **SpO_2_ <93%** | ● |  |  |  |
| **Fetal risk triggers** |  |  |  |  |
| **No fetal movements in last 12hr** | ● |  |  |  |
| **Heavy proteinuria ≥4+** | ● |  |  |  |
| **None of the 7 triggers and non-severe hypertension** (sBP 140-159mmHg) |  |  |  | ● |

** Unconscious women had to be severely hypertensive before receiving MgSO_4_; in the event that their unconsciousness was due to another cause unrelated to hypertension, such as obstetric sepsis or hypotension due to bleeding.*

*† MgSO_4_ was administered for eclampsia or severe pre-eclampsia, defined as hypertension that was either severe, or associated with stroke, abruption, or a high probability (≥25%) of an adverse maternal outcome within the next 48hr.*

*‡ Methyldopa was adminsitered only for severe hypertension.*

sBP=systolic blood pressure. SpO_2_=oxygen saturation by pulse oximetry.

***Table S3*: CLIP intervention**

|  | **Intervention**  **(n= 7930 pregnancies)**  **Median [IQR], n (%))** |
| --- | --- |
| Community engagement sessions | 4239 |
| Median community engagement sessions by cluster | 811 [465, 910] |
| Median community engagement participants by cluster | 3150 [2644, 4267] |
| CHWs trained | 79 |
| POM-guided CLIP contacts | 28,514 |
| Median POM-guided contacts/pregnancy | 6 [3, 8] |
| Antenatal | 4 [2, 6] |
| Postnatal | 2 [1, 300] |
| Emergency condition identified (and BP not taken) | 10/21 (47·6%) |
| BP measurement (all contacts) | 28287/28514 (99·2%) |
| Proteinuria measurement (of first and any hypertensive contact) | 4900/5015 (97·7%) |
| Complete visits that resulted in a recommendation | 27901/28514 (97·9%) |
| Pregnancies with ≥1 POM-guided CLIP contact | 4781/7930 (60·3%) |
| Complete visits | 28485/28514 (99·9%) |
| Pregnancies compliant with POM-guided CLIP contact frequency | 2783 (65·4%) |
| Pregnancies given methyldopa | 32 (0·7%) |
| Accepted | 22 (68·8%) |
| Pregnancies given IM magnesium sulphate | 31 (0·6%) |
| Accepted | 14 (45·2%) |
| Pregnancies referred to facility | 245 (5·1%) |
| Accepted | 168 (68·6%) |

CHW= community health worker. CEmOC=comprehensive emergency obstetric care. CLIP=Community-Level Interventions for Pre-eclampsia. IM=intramuscular. IQR=interquartile range. POM=PIERS On the Move.

***Table S4*: Sensitivity analyses**

|  | **Odds ratio (95% CI)** | **p-value** | **Adjusted** | **Imputed** |
| --- | --- | --- | --- | --- |
| Composite primary outcome* | 1.31 (0.70-2.45) | 0.40 | ✓ | ✓ |
| Unadjusted OR | 1.17 (0.66-2.07) | 0.59 | ✗ | ✓ |
| Complete postpartum follow-up | 1.29 (0.69-2.45) | 0.44 | ✓ | ✗ |
| Complete responses to all components of primary outcome | 1.29 (0.69, 2.40) | 0.42 | ✓ | ✗ |
| EDD + 3 weeks falls within trial timeline | 1.32 (0.70-2.47) | 0.45 | ✓ | ✓ |
| EDD + 3 weeks + 42 days falls within trial timeline | 1.32 (0.70-2.47) | 0.43 | ✓ | ✓ |
| Including only pregnancies with POM contacts | 1·21 (0·64, 2.27) | 0·56 | ✓ | ✓ |
| Baseline-adjusted OR | 0·93 (0·59, 1.46) | 0∙75 | ✓ | ✓ |
| Maternal mortality† | NA | NA | NA | NA |
| Maternal morbidity | 0.80 (0.49, 1.30) | 0.37 | ✓ | ✓ |
| Stillbirth and neonatal mortality | 1.29 (1.02, 1.64) | 0.03 | ✓ | ✓ |
| Neonatal morbidity | 0.48 (0.25, 0.93) | 0.03 | ✓ | ✓ |
| Cluster-level aggregate model | 0·97 (0·90, 1·05) | 0·45 | ✗ | ✗ |

OR=odds ratio. POM=PIERS On the Move. EDD=estimated date of delivery.

** Defined as one/more of maternal morbidity or mortality, stillbirth, neonatal mortality, or neonatal morbidity*

*† Number of cases insufficient to calculate the odds ratio.*
