# Supplementary material for: Community-level interventions for pre-eclampsia (CLIP) in Mozambique: A cluster randomised controlled trial
Source: Pregnancy Hypertens. 2020 Jul;21:96–105. doi: 10.1016/j.preghy.2020.05.006 (PMC7471842; doi:10.1016/j.preghy.2020.05.006)
Supplement: Supplementary data 4 — Figure S1. The CLIP intervention. [file mmc4.pdf]

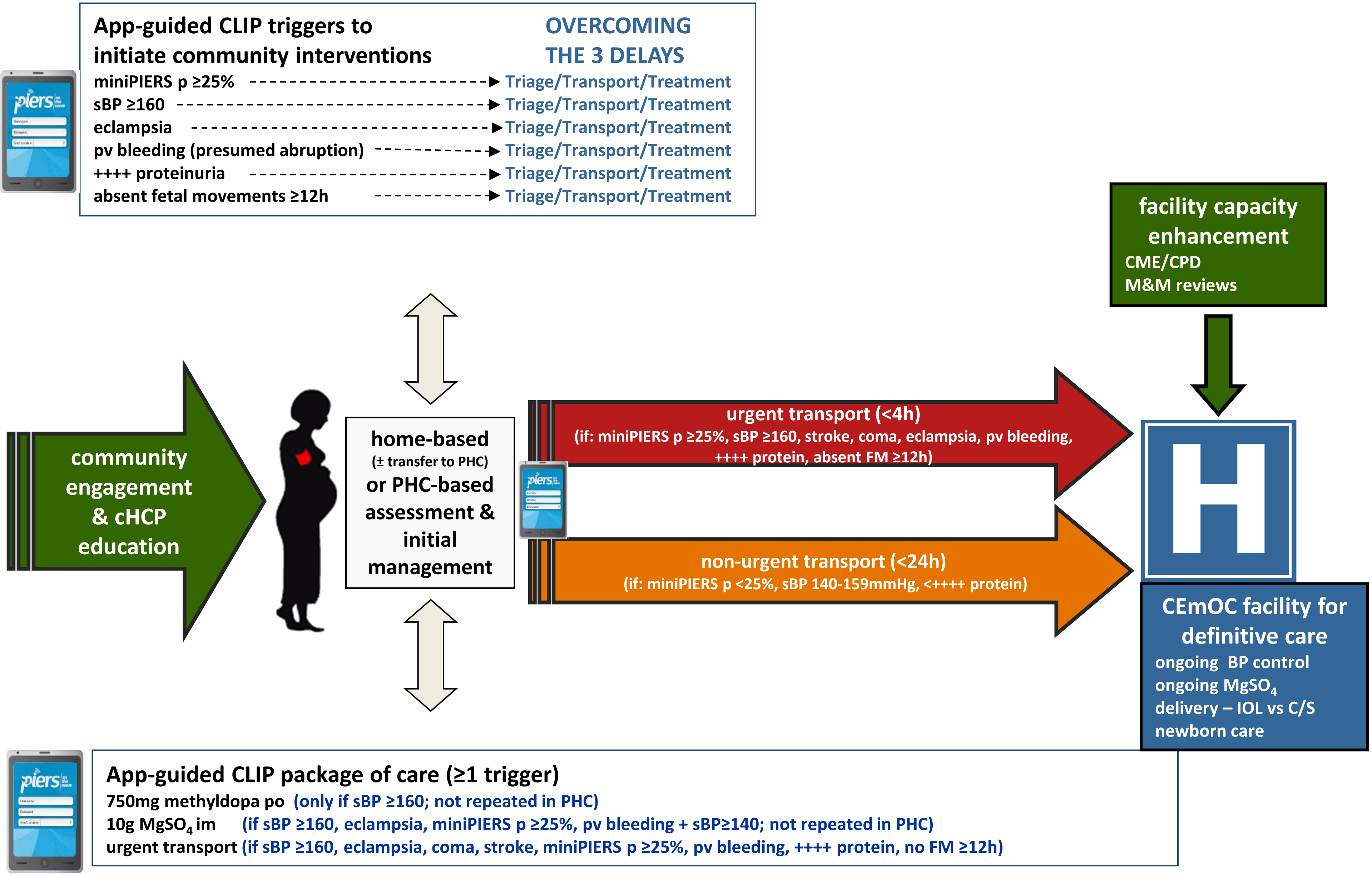

miniPIERS p= the probability of an adverse outcome using the miniPIERS (Pre-eclampsia Integrated Estimate of RiSk) model. sBP Systolic Blood Pressure. SI= Signal-Quality Index SpO2= Oxygen Saturation. pv= Vaginal. CME/CPD= Continuous Medical Education/ Continuous Professional Development. CEmOC= Comprehensive Emergency Obstetric Care MgSO4= Magnesium Sulphate. im= Intramuscular PHC= Primary Health Centre. cHCP= Communy Healh Care provider. FM= Fetal Movement
